# A Cross-sectional Study to Determine Prevalence of Obesity in High Income Group Colonies of Gwalior City

**DOI:** 10.4103/0970-0218.55287

**Published:** 2009-07

**Authors:** Ranjana Tiwari, Dhiraj Srivastava, Neeraj Gour

**Affiliations:** Department of PSM/Community Medicine, G.R Medical College, Gwalior (MP), India

**Keywords:** Body mass index, dietary profile, obesity, physical exercise

## Abstract

**Background:**

Obesity has become a major chronic disorder affecting the larger population more than any other disease in the world.

**Objectives:**

1) To determine the prevalence of obesity in both sexes in persons aged 30 years and above. 2) To determine the relationship of epidemiological determinants on the obesity status in the study subjects.

**Materials and Methods:**

The present study had been undertaken in literate high income group colonies of Gwalior city in which persons aged 30 years and above, in a family, were interviewed. A house-to-house survey method on a pre-designed, pre-tested structured questionnaire was used. Information regarding socio-demographic profile, eating habits and current health status were recorded. Anthropometric data regarding height, weight and blood pressure was also taken. The data was collected and analyzed using statistical software and chi square and proportional statistical test were applied.

**Results:**

The study showed that 34.4% of males and 31.3 % of females, both aged 30years and above were either obese or over weight. There was a statistically significant difference noted in the likening of fried food and fast food between obese and overweight persons and persons with normal body mass index.

**Conclusions:**

It can be concluded from the present study that obesity is a chronic illness. Early detection of it can prevent various complications associated with it. BMI plays a crucial role in its early detection as it is simple to calculate and can even detect the pre-obesity stage in time.

## Introduction

This has been a century of great changes. In the 21^st^ century, changes were noted not only in the science and technology but also in the life style of its inhabitants. Changes in the life style made life easier and marked the beginning of certain chronic ailments such as osteoarthritis, cardiovascular disorders, hypertension and obesity.(1)

Nowadays, obesity has become a chronic disorder affecting the larger population than any other disease in the world. It mostly affects the adult population but children and adolescent are also prone to develop obesity. According to the World Health Organization (WHO), nearly 20 to 40 % of adult population and 10 to 20% of children are affected by obesity.(2) Obesity, which made its presence felt first in the Northern Hemisphere, has now taken a pandemic look affecting practically almost all the countries of the globe. Obesity is not just limited to urban and affluent society but also affects the rural places and persons belonging to the lower socio-economic strata. Various epidemiological determinants have been responsible for the development of obesity, notably among them are Dietary habits, Physical Inactivity, Alcoholism, Smoking etc.

Body Mass Index (BMI) has been one of the easiest ways to determine the transition of a person from normal weight to obesity. It is simple to calculate and it categorizes a person as underweight, normal, overweight and obese with its stages.(2) Thus, BMI not only identifies obesity but also persons in pre-obese stages. So a screening program based on BMI would be helpful not only in identifying obese but also pre-obese persons so that timely measures could be taken for its correction, prevention and control persons and community as a whole.

This study was designed with the following objectives:

1. To determine the prevalence of obesity in both the sexes in persons aged 30 years and above.
2. To determine the relationship of epidemiological determinants with the obesity status in the study subjects.

## Materials and Methods

This is a field-based cross sectional study carried out in four high income group colonies (HIG) of Gwalior city of MP from Jan 2006 to May 2006 by students and staff of Dept. of Community Medicine, G.R Medical College, Gwalior. The four colonies selected had the highest land cost for the year 2004-05. (as per the office records of Municipal Corporation, Gwalior). The colonies also had a closely linked, similar socio-demographic profile, educational status and the cultural practices.

A study performa was developed, based on the socio-demographic profile, eating habits and present health status. Eating habits were enquired by asking the dietary profile of the participants regarding the vegetarian or mixed diet and also for fast and fried food diet. Mixed diet included both vegetarian and non vegetarian diet. The performa was pre-tested and necessary corrections were made accordingly.

For data collection, four teams were formed, each team comprising of four undergraduates students, one post graduate student and one faculty member from the Dept. of Community Medicine/PSM. Each team was provided with a similar set of instruments comprising of one bathroom scale weighing machine (accurate up to 0.5kg), measuring tape (maximum length 10m), a mercury sphygmomanometer and a stethoscope. The weighing machine and mercury sphygmomanometer were checked and corrected, if required, for zero error before the start of study. They were also checked and corrected, if required, after every 10^th^ reading during the study period.

The team members were given a day's training of approximately five hours in which each point of the performa was explained and discussed. Special emphasis was given to the method of recording of blood pressure, measurement of height and enquiring the details of dietary profile and type of exercise performed. Practical demonstrations were also made for these parameters to avoid the error during recording.

The houses in each selected colonies were numbered starting with the first house being closest to a fixed landmark in a colony. A house-to-house survey was undertaken to determine the number of persons aged 30 years or above. Houses which do not had persons aged 30 years or more were excluded from the study. The objectives of the study were explained to the participants and verbal consent was taken from them for active participation. A total of 1559 participants were listed in the study in which only 1438 gave the consent for participation while the remaining 121 participants included drop outs who could not be contacted even after the third visit to the house and those who did not gave the consent for active participation. A total of 482 houses were surveyed in which 652 households were contacted.

The information was recorded on a pre-designed, pre-tested and pre-structured questionnaire. For anthropometric measurements, a fixed algorithm was developed and used by all the teams.

The height of the participants was measured by asking them to stand barefoot by facing the back adjacent to the wall and keeping a scale straight on the head. A point was marked by the pencil on the wall. The participants were then asked to move and the length was measured using measuring tape in meters.

For calculation of the weight, the participants were asked to stand on the bathroom scale weighing machine, which was placed horizontally on a level surface and participants were asked to stand on it without any footwear and with minimum covered clothings.

The BP was recorded by first asking the participants to lie on bed and relax for 10 minutes. The systolic reading was recorded when the first sound was heard and then the diastolic reading was recorded when the sound disappears. Three such readings were recorded for both systolic and diastolic blood pressure and then the mean was calculated and noted down.

All the data was transferred to a computer and was double checked for any probable keyboard mistakes. Proportion and Chi Square test were applied for analysis using suitable statistical software.

## Results

A total of 1559 participants were included in the study, however, only 1438 subjects gave verbal consent for the participation in the study. Thus a total sample was restricted to 1438 only. Out of 1438 participants, 683 participants were males and 755 participants were females. The anthropometric variables of the study population was given in Table 1.

**Table 1:** Correlation between age and sex wise distribution in relation to weight, height and BMI

Age group in years	Male				Female			
		
	Number	Weight (SD) in cm	Height (SD) in kg	BMI (SD) Kg/m^2^	Number	Weight (SD) in cm	Height (SD) in kg	BMI (SD) Kg/m^2^
30-40	155	60.1	162.4	22.7	171	48.1	145.1	22.8
		(7.2)	(8.1)	(4.8)		(6.9)	(5.9)	(4.9)
40-50	147	61.8	161.9	23.57	189	53.8	145.5	25.4
		(6.7)	(7.9)	(5.1)		(6.6)	(6.2)	(4.7)
50-60	161	61.4	160.9	23.71	175	54.9	144.2	26.40
		(6.6)	(6.6)	(4.5)		(7.1)	(6.9)	(4.1)
60-70	138	59.6	161.1	22.96	149	49.2	145.7	23.1
		(6.6)	(7.1)	(4.8)		(6.6)	(6.2)	(4.8)
>70	82	58.1	159	22.98	71	44.2	142.5	21.1
		(5.9)	(6.2)	(3.7)		(5.1)	(5.9)	(3.7)
Total	683	60.2	161.06	23.1	755	50.04	144.6	23.76
		(6.6)	(7.1)	(4.4)		(6.4)	(6.2)	(4.4)

The mean height and weight of male participants was 161.06 cm (plus/minus 7.1 cm) and 60.2 kg (plus/minus 6.6 kg) respectively. Similarly the mean height and weight of female participants was 144.6 cm (plus/minus 6.2 cm) and 50.04 kg (plus/minus 6.4 kg) respectively.

The study had found that 34.4% of males and 31.3% females were either obese or overweight according to the WHO classification of BMI(2) [Table 2]. Most of the obese and overweight males belonged to age groups 50-60 years and females belonged to age group of greater than 70 years.

**Table 2:** Age and sex wise distribution in relation to different ranges of BMI

	Age in years	Number of participants	<18.5 kg/m^2^	18.5-24.9 kg/m^2^	25-29.9 kg/m^2^	30-34.9 kg/m^2^	35-39.9 kg/m^2^	>40 kg/m^2^
Male	30-40	155	15 (9.6)	92 (59.3)	32 (20.6)	11 (7.09)	4 (2.5)	10 (6.4)
	40-50	147	9 (6.1)	81 (55.1)	29 (19.7)	15 (10.2)	6 (4.08)	3 (2.04)
	50-60	161	4 (2.4)	91 (56.5)	35 (21.7)	19 (11.8)	9 (5.5)	3 (1.8)
	60-70	138	11 (7.9)	84 (60.8)	29 (21.01)	5 (3.6)	4 (2.8)	0
	>70	82	19 (23.1)	42 (51.2)	15 (18.2)	3 (3.6)	3 (3.6)	0
	Total	683	58 (8.4)	390 (57.1)	140 (20.4)	53 (7.7)	26 (3.8)	16 (2.3)
Female	30-40	171	25 (14.6)	101 (59.06)	27 (15.7)	17 (9.9)	1 (0.5)	0
	40-50	189	15 (7.9)	105 (55.5)	48 (25.3)	15 (7.9)	5 (2.6)	2 (1.05)
	50-60	175	13 (7.4)	111 (63.4)	24 (13.7)	18 (10.2)	7 (4)	1 (0.5)
	60-70	149	14 (9.3)	89 (59.7)	25 (16.7)	17 (11.4)	3 (2.01)	0
	>70	71	19 (26.7)	26 (36.6)	11 (15.4)	14 (19.7)	1 (1.4)	1 (1.4)
	Total	755	86 (11.3)	432 (57.2)	135 (17.8)	81 (10.7)	17 (2.2)	4 (0.5)

Figures in parenthesis are in percentage

A large number of obese and overweight males were involved in service sector i.e. government or private sector. These were followed by business men. However, most of the female participants were housewife's [Table 3].

**Table 3:** Showing the occupation of participants' sex wise

Occupation	Male		Female	
		
	Number	%	Number	%
Unemployed / House wife	21	3	695	92
Business	123	18	0	0
Service (Govt.)	184	27	0	0
Service (Pvt.)	293	43	45	6
Retired	62	9	15	2
Total	683	100	755	100

A relatively good number of obese and overweight participant both males and females donot exercise or were involved in mild physical exercises of less than or equal to five minutes or walking for about two km per day as per the WHO certeria for physical excerise which had been modified to suit the local needs. The female participants were more dormant in comparision to male participants [Table 4].

**Table 4:** Showing the exercising tendency of the obese and over weighted participants' sex wise

Exercise	Male		Female	
		
	Number	%	Number	%
No exercise	81	34.5	151	63.7
Mild exercise	93	39.5	72	30.3
Moderate exercise	49	20.8	14	6
Brisk exercise	12	5.2	0	0
Total	235	100	237	100

No significant difference was noted in the dietary profile of the participant as far as vegetarian and mixed diet is concerned (*P* > 0.05) [Table 5] but a significant difference was noted as far as liking for fried food and fast food was considered. (*P* < 0.05) [Table 6].

**Table 5:** Correlation of dietary pattern sex wise with cut of point of BMI

	Male		Female	
		
BMI Group	Vegetarian	Mixed	Vegetarian	Mixed
<24.9	236	221	297	221
>24.9	121	105	141	96
Total	357	326	438	317
*P* value	*P*>0.05(0.64018)		*P*>0.05(0.577182)	
	(X^2^=0.22) df-2		(X^2^=0.31) df-2	

**Table 6:** Correlation between liking for fried/fast food and sex, with cut of point of BMI

	Male		Female	
		
	Yes	No	Yes	No
BMI group				
<24.9	247	210	268	250
>24.9	144	82	148	89
Total	391	292	416	339
*P* value	P<0.05(0.016248)		P<0.05(0.006039)	
	(X^2^=5.78) df-2		(X^2^=7.54) df-2	

The study had found that as the BMI increases, there was a significant increase in Systolic Blood Pressure(according to JNC-V classification).(3) This increase in systolic BP was noted in both the sexes [Table 7].

**Table 7:** Correlation between BMI, sex wise, different ranges of systolic BP

	<130 mm Hg	130-140 mm Hg	140-160 mm Hg	160-180 mm Hg	>180 mm Hg	Total	*P* value
Male							
<18.5 Kg/m^2^	11 (18.9)	21 (36.2)	17 (29.3)	8 (13.7)	1 (1.7)	58	*P*<0.05 (0.0254) X^2^=34.1 df-20
18.5-24.9 Kg/m^2^	131 (32.8)	117 (29.3)	89 (22.3)	49 (12.2)	13 (3.2)	399	
25-29.9 Kg/m^2^	33 (23.5)	55 (39.2)	31 (22.1)	14 (10)	7 (5)	140	
30-34.9 Kg/m^2^	9 (16.9)	17 (32.07)	15 (28.3)	6 (11.3)	6 (11.3)	53	
35-39.9 Kg/m^2^	5 (19.2)	5 (19.2)	7 (26.9)	6 (23.07)	3 (11.5)	26	
>40 Kg/m^2^	0	3 (42.8)	2 (28.5)	2 (28.5)	0	7	
Total	189	218	161	85	30	683	
Female						
<18.5 Kg/m^2^	31 (36.04)	30 (34.8)	14 (16.2)	9 (10.4)	2 (2.3)	86	
18.5-24.9 Kg/m^2^	164 (37.9)	157 (36.3)	78 (18.05)	27 (6.2)	6 (1.3)	432	P<0.05 (0.0107) X^2^= 37.2 df-20
25-29.9 Kg/m^2^	54 (40)	38 (28.1)	32 (23.7)	8 (5.9)	3 (2.2)	135	
30-34.9 Kg/m^2^	32 (39.5)	17 (20.9)	19 (23.4)	7 (8.6)	6 (7.4)	81	
35-39.9 Kg/m^2^	4 (23.5)	5 (29.4)	4 (23.5)	3 (17.6)	1 (5.8)	17	
>40 Kg/m^2^	0	1 (25)	1 (25)	1 (25)	1 (25)	4	
Total	285	248	148	55	19	755	

Figures in parenthesis are in percentage

## Discussions

The present study had found that the prevalence of increase BMI is quite prevalent among adult population aged 30 years and above. The above study had found that males were taller (mean height 161.06 cm (plus/minus 7.1 cm)) and heavier (mean weight 60.2 kg (plus/minus 6.6 kg)) then females (mean height 144.6 cm (plus/minus 6.2 cm) and mean weight 50.04 kg (plus/minus 6.4 kg) respectively). Shukla *et al*, (4) Das *et al*.(5) also reported similar finding in their studies. The Average BMI of both male and female participants was 23.1 kg/m^2^ (plus/minus 4.4 kg/m^2^) and 23.7 kg/m^2^ (plus/minus 4.6 kg/m^2^) respectively.

The present study revealed that 34.4% of male participants and 31.3% of female participants were either obese or overweight (using greater than or equal to 25 kg/m^2^ cut of point, WHO 2000).(2) These rates are similar to the rates reported by Shukla *et al*.(4) - Male 19% and Female 30%, but was higher then rates quoted by Zargar *et al.*(6)-Male 7.01% and Female 23.69%. Asthana *et al*.,(7) and Mithu *et al*.(8) who also screened the prevalence of obesity in affluent female population found the prevalence of increased BMI in their study population which was 30.24% and 17.45% respectively.

The present study had depicted that as the age advances there is a slight increase in BMI with maximum prevalence among persons of age groups 50-60 years in males and in females more than 70 years. These age groups are also the age groups where maximum incidences of complications are associated with increase BMI.

It was also noted that majority of the male participants, obese and overweight, belonged to service sector (70%) either government or private followed by businessmen (18%), whereas majority of females were (92%) housewife. It may be attributed to the fact that these jobs profile required long hours of sitting or mild physical exercise.

The present study also showed that a sizable population of males (73.8 % (167/226)) and females (94 % (223/237)) do not exercise or do mild physical exercise. Khanan *et al*.(9) studied the attitude of UK Bangladeshi women and noticed that large fractions, 96% of obese and overweight women were not interested in physical exercise.

A comparison of the dietary profile of the two groups, BMI greater than 24.9 kg/m^2^ and BMI les than 24.9 kg/m^2^, no statistically significant difference, in both the sexes, was noted in Veg. or Mixed diet but statistically significant difference was noted when liking for fried and fast food was asked. Sinhababu A(10) who studied the dietary pattern among nursing students also reported that significant difference between obese and non obese students was there when liking for fast food and fried food was compared.

The study showed that as the BMI increases there is an increase in prevalence of raised Systolic BP. This is prevalent in both the sexes. Saha *et al*,(11) Gosh *et al*.,(12) Bell *et al*.(13) etc from different part of the country and around the globe also reported similar findings using various statistical test.

## Conclusions

It can be concluded from the present study that obesity and overweight are quite prevalent among adult population especially 30 years and above in both the sexes. BMI is a simple and effective way to screen obese and overweight persons residing in HIG colonies so that timely measures could be taken to prevent their progression and complications associated with it.

Measures to increase physical exercises both at home and at workplace could be undertaken using the behavior change communication strategy. Awareness regarding the impact of fried and fast food on health could be spread among general populations. Persons with BMI greater than 24.9 kg/m^2^ should be motivated to undergo regular screening of their BP.
